# Cophylogenetic interactions between marine viruses and eukaryotic picophytoplankton

**DOI:** 10.1186/1471-2148-14-59

**Published:** 2014-03-27

**Authors:** Laure Bellec, Camille Clerissi, Roseline Edern, Elodie Foulon, Nathalie Simon, Nigel Grimsley, Yves Desdevises

**Affiliations:** 1 Integrative Biology of Marine Organisms, Observatoire Océanologique, Sorbonne Universités, UPMC Univ Paris 06, UMR 7232, F-66650 Banyuls-sur-Mer, France; 2 Integrative Biology of Marine Organisms, Observatoire Océanologique, CNRS, UMR 7232, 66650 Banyuls-sur-Mer, France; 3 Marine Adaptation and Diversity, Station Biologique de Roscoff, Sorbonne Universités, Univ Paris 06, UMR 7144, F-29680 Roscoff, France; 4 Marine Adaptation and Diversity, Station Biologique de Roscoff, CNRS, UMR 7144, 29680 Roscoff, France

**Keywords:** Cophylogeny, Prasinovirus, Phycodnaviridae, Mamiellale, Chlorophyta, Specificity

## Abstract

**Background:**

Numerous studies have investigated cospeciation (or cophylogeny) in various host-symbiont systems, and different patterns were inferred, from strict cospeciation where symbiont phylogeny mirrors host phylogeny, to complete absence of correspondence between trees. The degree of cospeciation is generally linked to the level of host specificity in the symbiont species and the opportunity they have to switch hosts. In this study, we investigated cophylogeny for the first time in a microalgae-virus association in the open sea, where symbionts are believed to be highly host-specific but have wide opportunities to switch hosts. We studied prasinovirus-Mamiellales associations using 51 different viral strains infecting 22 host strains, selected from the characterisation and experimental testing of the specificities of 313 virus strains on 26 host strains.

**Results:**

All virus strains were restricted to their host genus, and most were species-specific, but some of them were able to infect different host species within a genus. Phylogenetic trees were reconstructed for viruses and their hosts, and their congruence was assessed based on these trees and the specificity data using different cophylogenetic methods, a topology-based approach, Jane, and a global congruence method, ParaFit. We found significant congruence between virus and host trees, but with a putatively complex evolutionary history.

**Conclusions:**

Mechanisms other than true cospeciation, such as host-switching, might explain a part of the data. It has been observed in a previous study on the same taxa that the genomic divergence between host pairs is larger than between their viruses. It implies that if cospeciation predominates in this algae-virus system, this would support the hypothesis that prasinoviruses evolve more slowly than their microalgal hosts, whereas host switching would imply that these viruses speciated more recently than the divergence of their host genera.

## Background

Viruses are recognized as important players in marine microbial ecosystems [1], but while the role of prokaryotic viruses (phages) has been widely appreciated in the last decades [2], our knowledge about viruses infecting the eukaryotic microbes and in particular microalgae, is much more recent [3,4]. Algal viruses, like aquatic phages, regulate the ecology and the evolution of their host populations via lysis and horizontal gene transfer [5], but they show high levels of host specificities [6-8]. In order to gain some understanding of oceanic ecosystems, it is thus important to analyse how viruses are transmitted from a host to another: are they mostly vertically transmitted, from ancestor to descendant, hence globally coevolving and cospeciating with their hosts with limited possibilities to switch to other host species, or can they easily colonize different host species, even phylogenetically distantly related? An understanding of the pattern of host-virus cospeciation, or cophylogeny (see [9,10]) is needed for comparing evolutionary rates based on molecular divergences (e.g. [11]), because it relies on the assumption of simultaneous speciation. Among the best-known viruses of planktonic eukaryotes are the phycodnaviruses (family Phycodnaviridae, [12]). Their similarity in structure (all of them are icosahedral particles enclosing double-stranded DNA) combined to the wide diversity of their hosts ("algae" that span most of the eukaryotic evolutionary tree), suggest that host switches have happened in the past. In any case, no detailed study of cophylogenetic interactions (i.e. at a macroevolutionary level) between algal viruses and their hosts has been carried out to date. The present work focuses on the host-virus association formed between prasinophytes, and more precisely, phytoplanktonic genera in the order Mamiellales, and their viruses, the prasinoviruses (see [13]). Mamiellales (class Mamiellophyceae, [14]), are an ecologically important group of marine picoeukaryotes that include three geographically widely distributed genera, *Micromonas*, *Ostreococcus* and *Bathycoccus*[15] (as well as the less represented genera *Mamiella*, *Mantoniella*, *Dolichomastix* and *Crustomastix*). The genomes of several species in the former three genera and their viruses have been entirely sequenced (see [11]). Numerous genetically diverse strains have been isolated [16-18], and probably encompass more species than those formally described so far (1 *Bathycoccus* species: *B. prasinos* Eikrem & Throndsen 1990, 1 *Micromonas* species: *M. pusilla* (Butcher) Manton and Parke 1960, and 3 *Ostreococcus* species: *O. tauri*[19], *O. lucimarinus*[20] and *O. mediterraneus*[21]). In *Micromonas*, Guillou et al. distinguished 3 major genetic clades (A, B and C) [16] while other studies [22-24] further distinguished sub-clades within clades A and B depicted in [16] suggesting that this genus contains at least 5 species. In *Ostreococcus*, Rodriguez et al. [17] have described four distinct clades clustering strains differing in their sensitivities to light (as well as temperature and nutrients [25], and also suggested they represent different species, as recently supported [21]. All of the *Bathycoccus* strains recorded so far form a more homogeneous evolutionary group [16], but recent work based on metagenomic analyses of natural samples suggested that this genus includes 2 to 3 different genotypes [26]. A similar picture is seen in prasinoviruses of the Mamiellophyceae, which form a monophyletic group [13]. Many genetically different strains have been characterized and form distinct clades according to the host species from which they were isolated [13,27]. Hence, these associations include numerous host and viral strains, but nothing is known to date about their joint macroevolutionary history, that is does the evolution of the viruses follow that of their hosts, which would be reflected by congruent phylogenetic trees?

We hereby investigate the cophylogenetic pattern in this host-virus system using molecular phylogenies, by experimentally assessing the specificity of many virus strains on several host strains, and we use dedicated numerical methods to assess the level of cospeciation. Understanding how hosts and viruses coevolve, taking into account the observed pattern of host specificity, is crucial for predicting the possibility of viral host-switching and for understanding speciation processes. For example, strict host specificity and limited capacities for viruses to switch hosts should be reflected in a strong cospeciation pattern, whereas strict host specificity with no cospeciation suggests a high speciation rate in viruses.

We show that these viruses are generally highly host-specific and display a significant, while complex, cophylogenetic pattern with their hosts. This may have important implications for the ecology and the dynamics of planktonic ecosystems.

## Results

### Specificity

**Figure 1:**
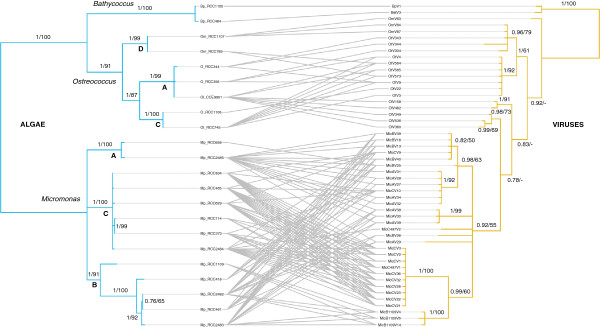
**Tanglegram depicting the pattern of infection of algal hosts by viruses.** Trees were reconstructed from DNA sequences (partial DNA polymerase gene for viruses, concatenated 18S rDNA and ITS2 for hosts) using Bayesian Inference (BI) and Maximum Likelihood (ML), and the BI tree is presented. Numbers indicate clade support as posterior probabilities (BI, from the analysis of translated sequences)/bootstrap values in% (ML, - indicates numbers < 50). Letters refer to host clades.

Experimental tests of host-specificity suggest that virus strains are specific to their host genus (Table 1), and infect in majority host species from the same clade (Table 1 and Figure 1). However, a number of strains display a cross-clade specificity (14 out of 31 (45.2%) in *Micromonas* viruses, and 4 out of 18 (22.2%) in *Ostreococcus* viruses). Host range (i.e. specificity) varies from 1 to 6 in virus strains studied here, and 18 viruses out of 51 (35.3%) are strict specialists, infecting only 1 host strain.

**Table 1:** **Host specificity of viruses (Ot stands for *Ostreococcus tauri*, Ol is *O. lucimarinus*, Om is *O. mediterraneus*, Osp is *Ostreococcus *** **sp., and Bp is *B. prasinos*). – no lysis; □ lysis (isolate from this host); ■ lysis**

**Species**	***Micromonas pusilla***													**Ot**	**Ol**	**Osp.**			**Om**		**Bp**	
Clade	A	A	B	B	B	B	B	C	C	C	C	C	C	C	A	A	A	C	D	D		
RCC no.	2485	658	2482	2483	418	461	1109	2484	834	629	465	373	114	745		356	344	1108	789	1107	1105	464
MicAV31	□	-	-	-	-	-	-	-	-	-	-	-	-	-	-	-	-	-	-	-	-	-
MicAV32	□	-	-	-	-		-	-	-	-	-	-	-	-	-	-	-	-	-	-	-	-
MicAV27	□	-	-	-	-	■	-	-	-	-	-	-	-	-	-	-	-	-	-	-	-	-
MicAV28	□	-	-	-	-	■	-	-	-	-	-	-	-	-	-	-	-	-	-	-	-	-
MicAV34	□	-	-	-	-	■	-	-	-	-	-	-	-	-	-	-	-	-	-	-	-	-
MicAV29	□	-	■	-	-	■	-	-	-	-	-	-	-	-	-	-	-	-	-	-	-	-
MicAV30	□	-	■	■	-	-	-	-	-	-	-	-	-	-	-	-	-	-	-	-	-	-
MicAV38	□	-	■	■	-	■	-	-	-	-	-	-	-	-	-	-	-	-	-	-	-	-
MicAV39	□	-	■	■	-	-	-	-	-	-	-	-	-	-	-	-	-	-	-	-	-	-
MicBV26	-	-	□	■	-	-	-	-	-	-	-	-	-	-	-	-	-	-	-	-	-	-
MicBV16	■	-	□	■	-	-	-	-	-	-	-	-	-	-	-	-	-	-	-	-	-	-
MicBV13	■	-	□	■	■	-	-	-	-	-	-	-	-	-	-	-	-	-	-	-	-	-
MicBV40	■	-	□	■	■	-	-	-	-	-	-	-	-	-	-	-	-	-	-	-	-	-
MicBV39	■	-	□	■	-	■	-	-	-	-	-	-	-	-	-	-	-	-	-	-	-	-
MicBV25	■	■	□	■	■		-	-	-	-	-	-	-	-	-	-	-	-	-	-	-	-
MicB1109V4	-	-	■	■	-	-	□	-	-	-	-	-	-	-	-	-	-	-	-	-	-	-
MicB1109V14	-	-	■	■	-	-	□	-	-	-	-	-	-	-	-	-	-	-	-	-	-	-
MicB1109V6	-	-	■	■	-	-	□	-	-	-	-	-	-	-	-	-	-	-	-	-	-	-
MicC497V1	-	-	-	-	-	-	-	□	■	■	■	-	■	-	-	-	-	-	-	-	-	-
MicC497V2	-	-	-	-	-	-	-	□	-	-	-	-	-	-	-	-	-	-	-	-	-	-
MicCV1	-	-	-	-	-	-	-	■	□	-	■		-	-	-	-	-	-	-	-	-	-
MicCV36	-	-	-	-	-	-	-	■	□	-	■	■	-	-	-	-	-	-	-	-	-	-
MicCV2	-	-	-	-	-	-	-	■	□	■	■	■	-	-	-	-	-	-	-	-	-	-
MicCV21	-	-	-	-	-	-	-	■	□	■	■	■	-	-	-	-	-	-	-	-	-	-
MicCV28	-	-	-	-	-	-	-	■	□	■	■	■	-	-	-	-	-	-	-	-	-	-
MicCV32	-	-	-	-	-	-	-	■	□	■	■	■	-	-	-	-	-	-	-	-	-	-
MicCV23	-	-	-	-	-	-	-	■	□	■	■		■	-	-	-	-	-	-	-	-	-
MicCV22	-	-	-	-	-	-	-	■	□	■	■	■	■	-	-	-	-	-	-	-	-	-
MicCV3	-	-	-	-	-	-	-	■	□	■	■	-	■	-	-	-	-	-	-	-	-	-
MicCV9	-	-	-	■	-	-	-	■	□	■	■	-	-	-	-	-	-	-	-	-	-	-
MicCV10	-	-	-	-	■	-	-	■	□	■	■	■	-	-	-	-	-	-	-	-	-	-
OlV158	-	-	-	-	-	-	-	-	-	-	-	-	-	-	□	-	-	-	-	-	-	-
OlV349	-	-	-	-	-	-	-	-	-	-	-	-	-	-	□	-	-	-	-	-	-	-
OlV360	-	-	-	-	-	-	-	-	-	-	-	-	-	-	□	-	-	-	-	-	-	-
OlV462	-	-	-	-	-	-	-	-	-	-	-	-	-	-	□	-	-	-	-	-	-	-
OlV536	-	-	-	-	-	-	-	-	-	-	-	-	-	-	□	-	-	-	-	-	-	-
OtV3	-	-	-	-	-	-	-	-	-	-	-	-	-	□	-	-	-	-	-	-	-	-
OtV4	-	-	-	-	-	-	-	-	-	-	-	-	-	□	-	■	-	-	-	-	-	-
OtV9	-	-	-	-	-	-	-	-	-	-	-	-	-	□	-	■	■	-	-	-	-	-
OtV564	-	-	-	-	-	-	-	-	-	-	-	-	-	□	■	-	-	-	■	-	-	-
OtV565	-	-	-	-	-	-	-	-	-	-	-	-	-	□	■	-	-	-	-	-	-	-
OtV573	-	-	-	-	-	-	-	-	-	-	-	-	-	□	-	-	-	-	■	-	-	-
OtV22	-	-	-	-	-	-	-	-	-	-	-	-	-	-	-	-	-	□	-	-	-	-
OtV343	-	-	-	-	-	-	-	-	-	-	-	-	-	-	-	-	□	-	-	-	-	-
OtV344	-	-	-	-	-	-	-	-	-	-	-	-	-	-	-	-	□	-	-	-	-	-
OtV304	-	-	-	-	-	-	-	-	-	-	-	-	-	-	-	□	-	-	-	-	-	-
OmV63	-	-	-	-	-	-	-	-	-	-	-	-	-	-	-	-	-	-	-	□	-	-
OmV64	-	-	-	-	-	-	-	-	-	-	-	-	-	-	-	-	-	-	-	□	-	-
OmV67	-	-	-	-	-	-	-	-	-	-	-	-	-	-	-	-	-	-	-	□	-	-
BpV1	-	-	-	-	-	-	-	-	-	-	-	-	-	-	-	-	-	-	-	-	□	-
BatV3	-	-	-	-	-	-	-	-	-	-	-	-	-	-	-	-	-	-	-	-	-	□

The susceptibility of host strains to viruses, i.e. the number of virus strains that can infect a given host strain, varies from 1 to 14. The most highly susceptible strains are found in *Micromonas*.

### Phylogeny

Sequences ranged between 609 and 624 bp (DNA polymerase gene or dpo) for viruses and 1996 and 2238 bp (rDNA 18S (1620–1635 bp) + ITS2 (366–607 bp)) for hosts. Respective alignment lengths were 624 bp (208 AA) and 2289 bp. Unpublished sequences were deposited in GenBank (Accession numbers, sequence lengths, strain names, geographical origin, and host culture for isolation are given in Tables 2 and 3).

**Table 2:** Host strains used in this study (RCC = Roscoff Culture Collection)

**Host species**	**Clade**	**Strain code in RCC**	**Alternative strain code (other culture collections)**	**Isolation site**	**Isolation date (dd-mm-yyyy)**	**Reference and/or accession number**
Mp	A	2485		Atlantic Ocean	11-07-1980	This study, KF501024
				(38°42*'*N, 72°22*'*W)		
Mp	A	658	CS-170	Pacific Ocean, West Australia	01-01-1982	This study, KF501030
Mp	B	2482		Mediterranean Sea, Italy	08-04-1993	This study, KF501032
Mp	B	2483		Mediterranean Sea, Italy	08-01-1997	This study, KF501033
Mp	B	418		English Channel	14-06-2001	This study, KF501026
				(48°37*'*N, 3°51*'*W)		
Mp	B	461		English Channel	14-06-2001	This study, KF501027
				(48°37*'*N, 3°51*'*W)		
Mp	B	1109		Mediterranean Sea, Leucate lagoon	28-07-2006	This study, KF501031
				(42°48*'*N, 3°1*'*E)		
Mp	C	2484		Mediterranean Sea, Spain	18-04-2002	This study, KF501034
				(41°43*'*N, 3°33*'*E)		
Mp	C	834	CCMP1545, PLY 27	English Channel	13-04-1950	[28]; AY954994
				(50°36*'*N, 3°57*'*W)		
Mp	C	629		North Sea, Germany	17-01-2001	This study, KF501018
				(54°11*'*N, 7°54*'*E)		
Mp	C	465		English Channel	13-06-2001	This study, KF501028
				(48°37*'*N, 4°17*'*W)		
Mp	C	373		Baltic Sea, Skagerrak	03-01-2001	This study, KF501025
				(58°11*'*N, 9°6*'*E)		
Mp	C	114	CCMP490	Atlantic Ocean, USA	18-06-1964	[22], AY955004
				(41°31*'*N, 70°40*'*W)		
Ot	C	745		Mediterranean Sea, Thau lagoon	03-05-1995	[19], CAID01000012
				(43°24*'*N, 3°36*'*E)		
Ol	A		CCE9901, CCMP2972	North Pacific, California	01-01-1999	[20], AY329636
				(32°90*'*N, 117°25*'*W)		
O	A	344		English Channel	04-12-2000	[16], AY425307 (18S); [17], AY586736 (ITS)
				(48°45′N, 3°57′W)		
O	A	356		North Atlantic, Morocco	09-12-1999	[16], AY425308 (18S); [17], AY586740 (ITS)
				(30°8*'*N, 10°3*'*W)		
O	C	1108		Mediterranean Sea, Banyuls Bay	01-02-2006	[18]; GQ426332
				(42°29*'*N, 3°8*'*E)		
Om	D	789		Mediterranean Sea, Spain	28-02-2001	[16], AY425313 (18S); This study, [17], AY586745 (ITS)
				(41°43*'*N, 3°33*'*E)		
Om	D	1107		Mediterranean Sea, (43°3*'*N, 2°59*'*E)	01-01-2006	[21], JN862902
Bp		1105		Mediterranean Sea, Banyuls Bay	01-01-2006	[29], JX625115
				(42°29*'*N, 3°8*'*E)		
Bp		464		English Channel	09-07-2000	This study, KF501036
				(48°45*'*N, 3°57*'*W)		

Mp = *Micromonas pusilla*, Ot = *Ostreococcus tauri*, O = *Ostreococcus* sp, Ol = *Ostreococcus lucimarinus*, Om = *Ostreococcus mediterraneus*, Bp = *Bathycoccus prasinos*. RCC2482, 2483, 2484 and 2485 are clonal strains isolated from RCC828, RCC829, RCC497 and RCC 451, respectively.

**Table 3:** Data on virus strains used in this study (RCC: Roscoff Culture Collection)

**Strain name in RCC**	**Original strain name**	**Isolation date (dd-mm-yyyy)**	**Isolation site**	**Reference and/or accession number**
2066	MicAV27	10-11-2009	SOMLIT-Astan (48°46*'* 18*''* N, 3°58*'* 6*''*W)	[8], KF378571
2067	MicAV28	10-11-2009	SOMLIT-Astan (48°46*'* 18*''* N, 3°58*'* 6*''*W)	This study, KF500985
2068	MicAV29	10-11-2009	SOMLIT-Astan (48°46*'* 18*''* N, 3°58*'* 6*''*W)	[8], KF378572
2069	MicAV30	10-11-2009	Ilot St-Anne (48°41*'*17*''*N, 3°57*'*27*''*W)	[8], KF378573
2070	MicAV31	11-11-2009	Ilot St-Anne (48°41*'*17*''*N, 3°57*'*27*''*W)	This study, KF500986
2071	MicAV32	11-11-2009	Ilot St-Anne (48°41*'*17*''*N, 3°57*'*27*''*W)	This study, KF500987
2073	MicAV34	12-11-2009	Ilot St-Anne (48°41*'*17*''*N, 3°57*'*27*''*W)	This study, KF500988
2075	MicAV38	12-10-2009	SOMLIT-Astan (48°46*'* 18*''* N, 3°58*'* 6*''*W)	This study, KF500989
2076	MicAV39	12-10-2009	SOMLIT-Astan (48°46*'* 18*''* N, 3°58*'* 6*''*W)	This study, KF500990
2082	MicBV13	20-03-2009	SOMLIT-Astan (48°46*'* 18*''* N, 3°58*'* 6*''*W)	This study, KF500993
2085	MicBV16	20-03-2009	SOMLIT-Astan (48°46*'* 18*''* N, 3°58*'* 6*''*W)	This study, KF500994
2093	MicBV25	18-05-2009	SOMLIT-Astan (48°46*'* 18*''* N, 3°58*'* 6*''*W)	This study, KF500995
2094	MicBV26	18-05-2009	SOMLIT-Astan (48°46*'* 18*''* N, 3°58*'* 6*''*W)	[8], KF378576
2099	MicBV39	16-04-2009	SOMLIT-Astan (48°46*'* 18*''* N, 3°58*'* 6*''*W)	[8], KF378578
2100	MicBV40	16-04-2009	SOMLIT-Astan (48°46*'* 18*''* N, 3°58*'* 6*''*W)	This study, KF500996
2199	MicB1109V4	28-09-2009	SOMLIT-Astan (48°46*'* 18*''* N, 3°58*'* 6*''*W)	This study, KF500991
	MicB1109V6	28-09-2009	SOMLIT-Astan (48°46*'* 18*''* N, 3°58*'* 6*''*W)	This study, KF500992
2206	MicB1109V14	28-09-2009	SOMLIT-Astan (48°46*'* 18*''* N, 3°58*'* 6*''*W)	[8], KF378564
2125	MicCV1	05-02-2009	SOMLIT-Astan (48°46*'* 18*''* N, 3°58*'* 6*''*W)	This study, KF500997
2126	MicCV2	05-02-2009	SOMLIT-Astan (48°46*'* 18*''* N, 3°58*'* 6*''*W)	This study, KF500998
2127	MicCV3	05-02-2009	SOMLIT-Astan (48°46*'* 18*''* N, 3°58*'* 6*''*W)	This study, KF500999
2131	MicCV9	03-04-2009	SOMLIT-Astan (48°46*'* 18*''* N, 3°58*'* 6*''*W)	[8], KF378580
2132	MicCV10	03-04-2009	SOMLIT-Astan (48°46*'* 18*''* N, 3°58*'* 6*''*W)	This study, KF501000
2135	MicCV21	04-05-2009	SOMLIT-Astan (48°46*'* 18*''* N, 3°58*'* 6*''*W)	This study, KF501001
2136	MicCV22	04-05-2009	SOMLIT-Astan (48°46*'* 18*''* N, 3°58*'* 6*''*W)	This study, KF501002
2137	MicCV23	04-05-2009	SOMLIT-Astan (48°46*'* 18*''* N, 3°58*'* 6*''*W)	This study, KF501003
2142	MicCV28	18-05-2009	SOMLIT-Astan (48°46*'* 18*''* N, 3°58*'* 6*''*W)	This study, KF501004
2146	MicCV32	02-06-2009	SOMLIT-Astan (48°46*'* 18*''* N, 3°58*'* 6*''*W)	[8], KF378579
2150	MicCV36	14-08-2009	SOMLIT-Astan (48°46*'* 18*''* N, 3°58*'* 6*''*W)	This study, KF501005
2167	MicC497V1	18-05-2009	SOMLIT-Astan (48°46*'* 18*''* N, 3°58*'* 6*''*W)	[8], KF378567
2168	MicC497V2	18-05-2009	SOMLIT-Astan (48°46*'* 18*''* N, 3°58*'* 6*''*W)	[8], KF378566
	OlV158	01-16-2008	Mediterranean Sea, Leucate lagoon (42°48*'*N, 3°1*''*E)	[27], GQ412099
	OlV349	09-26-2008	English Channel (48°45*'*N, 3°57*'*W)	[27], GQ412082
	OlV360	10-30-2008	South Pacific, Chili (36°32*'*S, 72°56*'*W)	[27], GQ412085
	OlV462	09-26-2008	Mediterranean Sea, Banyuls Bay (42°29*'*N, 3°8*'*E)	[27], GQ412091
	OlV536	10-20-2008	English Channel (48°45*'*N, 3°57*'*W)	[27], GQ412096
	OtV3	01-24-2006	Mediterranean Sea, La Palme lagoon (42°57*'*18.04*''*N, 3°0*'*3.56*''*E)	[13], FJ267504
	OtV4	01-24-2006	Mediterranean Sea, La Palme lagoon (42°57*'*18.04*''*N, 3°0*'*3.56*''*E)	This study, KF501006
	OtV9	13-02-2006	Mediterranean Sea, Thau lagoon (43°24*'*N, 03°36*'*E)	[30], JN225859
	OtV22	04-20-2006	Mediterranean Sea, Bages lagoon	[13], FJ267497
	OtV304	08-06-2008	Mediterranean Sea, Leucate lagoon (42°48*'*N, 3°1*'*E)	This study, KF501007
	OtV343	09-26-2008	Mediterranean Sea, Banyuls Bay (42°29*'*N, 3°8*'*E)	This study, KF501008
	OtV344	09-26-2008	Mediterranean Sea, Banyuls Bay (42°29*'*N, 3°8*'*E)	This study, KF501009
	OtV564	27-03-2009	Mediterranean Sea, La Palme lagoon (42°57*'*18.04*''*N, 3°0*'*3.56*''*E)	This study, KF501010
	OtV565	27-03-2009	Mediterranean Sea, La Palme lagoon (42°57*'*18.04*''*N, 3°0*'*3.56*''*E)	This study, KF501011
	OtV573	27-03-2009	Mediterranean Sea, La Palme lagoon (42°57*'*18.04*''*N, 3°0*'*3.56*''*E)	This study, KF501012
	OmV63	01-31-2007	Mediterranean Sea, La Palme lagoon (42°57*'*18.04*''*N, 3°0*'*3.56*''*E)	[13], FJ267501
	OmV64	01-31-2007	Mediterranean Sea, La Palme lagoon (42°57*'*18.04*''*N, 3°0*'*3.56*''*E)	[13], FJ267502
	OmV67	06-01-2007	Mediterranean Sea, Leucate lagoon (42°48*'*N, 3°1*'*E)	[13], FJ267500
	BpV1	10-31-2007	Mediterranean Sea, Banyuls Bay (42°27*'*N, 3°32*'*E)	[11], NC_014765
2211	BatV3	04-04-2009	English Channel (48°37*'*N, 4°17*'*W)	This study, KF501013

Evolutionary models selected with jModelTest were Tamura-Nei 93 + I + G (accounting for rate heterogeneity across sites via a Gamma distribution with a 0.327 alpha parameter and including 41% of invariant sites) for hosts (18S + ITS), and CpREV + G + F (using observed AA frequencies, and a 0.78 alpha parameter) for the alignment of virus protein sequences.

Whatever the methods (Bayesian inference (BI) or maximum likelihood (ML)) and datasets (DNA or AA for viruses), phylogenetic trees were the same for hosts and very similar for viruses, therefore only BI trees are presented (Figure 1. These trees and associated alignments were deposited in TreeBASE at the URL http://purl.org/phylo/treebase/phylows/study/TB2:S15522). The host phylogeny obtained is clearly coherent with the phylogeny of Mamiellophyceae published by [14] where *Ostreococcus* and *Bathycoccus* form the Bathycoccaceae and *Micromonas* strains cluster separately. Three distinct clades are observed in *Ostreococcus*, clustering strains in the clades A, C and D defined by [17] (no strain from clade B was included in the present analysis). Three well-supported clades are also found in *Micromonas. Bathycoccus* is much more homogeneous, and the only two strains retained are closely related. However, the 18S sequence from RCC1105 (*Bathycoccus*) included an intron that was removed for the phylogenetic analysis.

The phylogenetic tree of viral strains (Figure 1) suggests that viruses from *Ostreococcus* and *Micromonas* form a paraphyletic group, i.e. viruses from *Micromonas* are derived from *Ostreococcus* viruses. However, the *Micromonas* virus clade is strongly supported and contains several well-defined clades.

### Cophylogeny

The global congruence between trees using the distance-based approach ParaFit was highly significant (P = 0.001, see Table 4 for all results on cophylogenetic analyses), as were all the 138 individual host-virus links (each P-value is below 0.05). The event-based analysis with Jane, taking tree topologies into account, also yielded a highly significant global congruence between host and virus phylogenetic trees (P = 0.001), confirming the ParaFit analysis. The complexity of this host-symbiont system precludes the establishment of any intelligible coevolutionary scenario. Because of this complexity, we performed three partial analyses in addition to the full dataset: only *Micromonas* strains and their viruses, *Bathycoccus* and *Ostreococcus* strains and their viruses, and only *Ostreococcus* strains and their viruses. Analyses with ParaFit gave a significant global congruence (P = 0.001) between host and parasite trees for the *Micromonas* dataset (78/111 significant links) and the *Bathycoccus* and *Ostreococcus* dataset (12/26 significant links) but some individual host-virus links were found non-significant. For the *Ostreococcus* dataset, the global fit was not significant (P = 0.11; 1/25 significant links). In Jane analyses, the global congruence was significant for the *Micromonas* dataset (P = 0.001), but not for the *Bathycoccus*-*Ostreococcus* dataset (P = 0.1) and the *Ostreococcus* dataset (P = 0.46). Note that the results were similar with the trees obtained from BI or ML trees, and using a virus tree where the monophyly of the viruses of *Micromonas*, *Ostreococcus* and *Bathycoccus* were each enforced (built with the same method and parameters, and not significantly different from the tree on Figure 1 (Kishino-Hasegawa test: P = 0.330; Shimodeira-Hasegawa test: P = 0.160. Both tests were performed in PAUP with distributions generated from 1000 boostrap replicates by the resampling estimated log-likelihood method using a fully optimized model)).

**Table 4:** Results of the cophylogenetic analyses with ParaFit ("links" refer to individual host-virus associations) and Jane (costs for individual events: Cospeciation = 0, Duplication = 1, Host-switch = 2, Loss = 1, Failure to diverge = 1)

	**ParaFit**		**Jane**
	**P-value for global fit**	**Number of significant links/total**	**Global cost (P-value)**
All	0.001	138/138	236 (0.001)
Micro	0.01	78/111	183 (0.001)
Bathy-Ostreo	0.001	12/26	41 (0.1)

"All" refers to the full dataset, "Micro" to the association between only *Micromonas* hosts and their viruses, "Bathy-Ostreo" to *Bathycoccus* and *Ostreococcus* hosts and their viruses. All statistical tests were performed with 999 permutations.

## Discussion

Three main results emerge from the present study: (1) prasinoviruses are specific to their host genus (2); within a genus, their viruses are generally specific to a clade (i.e. they can infect different host strains that belong to the same clade) (3); the cophylogenetic analysis using ParaFit and Jane revealed significant patterns of associations between host and virus phylogenetic trees and then suggests the existence of a common macroevolutionary scenario between Mamiellophyceae and their viruses.

While viruses can often infect several host species or genetic clades, a phylogenetic specificity is nevertheless clearly observed, i.e. prasinoviruses tend to infect related host strains, as observed in *Ostreococcus* strains and their viruses [8]. This has also been previously observed in *Micromonas* viruses [31,32], and other virus-microalgae associations [4]. In the absence of studies determining the species of most of the host strains used, host clade is the best proxy we currently have for putative host species or ecotype [17,21,22]. By doing so, we observe that most Prasinovirus strains are species-specific, with the more generalist viruses tending to infect more related host species (see Figure 1 and Table 1). Given that high dispersion of hosts and viruses [27] allow them to enter in contact, it is likely that there is no ecological barrier to host switch. This suggests that mechanistic processes preclude viruses from infecting distantly related host species. Such inner structural limiting factors were also observed in the related chloroviruses [33]. We hypothesize that intrinsic factors affecting specificity should exist otherwise large host ranges would be common, as they would allow viruses to maintain themselves even when the populations of some of their host species are subjected to fluctuations, which is not observed. This is especially relevant, given that low density populations of host Mamiellales are often found in oligotrophic environments [34,35].

Some host strains (e.g. *Micromonas* RCC2484 or *Ostreococcus tauri* RCC745) are susceptible to a wider range of viruses than others (e.g. *Micromonas* RCC804 or *Ostreococcus* RCC1108). This may be related to lower resistance of these Mamiellale strains to viruses, as previously observed by Thomas et al. [30], who showed experimentally that resistance to a given viral strain was associated to increased sensitivity to other viral strains as well as to loss in fitness (i.e. slower growth) compared to susceptible host lines, and a balance between resistance or susceptibility was observed in culture, depending on the partners present. This trade-off, combined with the host range of particular viruses, results in the complex pattern of specificity/sensitivity observed in the present study. The highest sensitivity observed here in *Micromonas* perhaps reflects the higher number of host and virus strains found in this genus.

In the last three decades, many studies investigating cospeciation in host-symbiont systems e.g. [36-40], see [9] have been published, and report various analytical methods [40-46]. However, while a number of host-virus system have been studied to date in a cophylogenetic framework [47-50], to our best knowledge none were carried out on an aquatic association. In a vast aquatic environment, such as the open sea, the barriers to host switching can be seen as generally weaker than in a structured terrestrial ecosystem where local adaptation can occur more easily [51], especially given the wide dispersal of hosts and viruses in the marine ecosystem. Any cospeciation signal is thus more likely due to close adaptation to the host than to the impossibility to switch hosts.

A significant global signal of cospeciation was found with all methods used, suggesting that Prasinovirus evolution is in part driven by the evolution of their hosts, or at least that related viruses tend to use related hosts. When partitioning the dataset into *Micromonas* strains and their viruses, *Bathycoccus*-*Ostreococcus* strains and their viruses, and *Ostreococcus* strains and their viruses, results were slightly different. The global fit analysis with ParaFit found a significant congruence in all cases, while the event-based analysis with Jane found a non-significant congruence for the *Bathycoccus*-*Ostreococcus* and the *Ostreococcus* datasets. This lack of significant signal may reflect a genuine lack of cophylogenetic signal, or may be due to a lower statistical power with less data (because the null hypothesis is the absence of congruence). The differing results obtained by ParaFit and Jane for *Bathycoccus*-*Ostreococcus* and *Ostreococcus* only might also arise because the genetic distances (used by ParaFit) do not always correlate with phylogenetic (patristic) distances as used in Jane. Thus genetically close viruses tend to colonize close hosts but this may not be always the case at a phylogenetic point of view supporting the hypothesis that viruses can switch to different, but not too distantly related, host strains. The global significance observed for the complete dataset with all methods confirms the genus-specificity of viruses: *Bathycoccus*, *Ostreococcus* and *Micromonas* have their own viruses that do not cross the genus boundaries with detectable frequencies. Within each genus, even if viruses tend to be clade-specific, several strains possess a wide intrageneric, and probably interspecific, host range. This is especially true in *Micromonas* viruses where several strains can infect hosts from the three clades, while this pattern is much less frequent in *Ostreococcus* viruses. However, this is not strong enough to break the significant cophylogenetic congruence between *Micromonas* strains and their viruses (P = 0.01), while between *Ostreococcus*-*Bathycoccus* and their viruses the cophylogenetic signal is not significant, as well as between *Ostreococcus* and their viruses. The different results obtained with ParaFit and Jane for the *Ostreococcus*-*Bathycoccus* dataset may in part be due to the different ways these methods works: ParaFit relies only on distances and the influence of the tree topology on the outcome is far less important than in a method such as Jane. However, this issue exists for the *Micromonas* as well as for the *Bathycoccus*-*Ostreococcus* and *Ostreococcus* datasets (which contain less taxa, then less data, decreasing the statistical power), and the cophylogenetic congruence in the *Bathycoccus*-*Ostreococcus* dataset is only slightly below the significance threshold with Jane. Duplication and sorting probably also play a role here to explain the lack of topological congruence between trees. However, while viruses seems currently unable to switch from a genus to another, the absence of match between host and virus phylogenetic trees at the genus level suggests an early host-switch from *Bathycoccus* to *Micromonas* whose colonization by prasinoviruses would then be more recent. That could explain the more general pattern of association between *Micromonas* strains and their viruses with strains displaying a cross-clade specificity.

In most previous cophylogenetic analyses on host-virus systems, a significant cospeciation signal was found [47,49,50,52-64]. However complex cophylogenetic histories were often estimated, mixing codivergence with host-switches, duplication and losses [50] and in some cases, no significant cospeciation signal was inferred [48,49,61,65,66]. The general tendency is however that virus evolution is strongly linked to that of their hosts, which is coherent with the results found in the present study, in a totally different environment to those previously investigated for viruses of eukaryotes. The tendency to cospeciate with hosts is thus probably due to intrinsic features of viruses (e.g. mechanistic causes such as molecular characteristics constraining the use of specific hosts) rather than to ecological barriers.

The presence of a cospeciation signal does not necessarily imply real cospeciation, i.e. a significant amount of concomitant speciation in hosts and their parasites. For example, a host switch to a sister host species followed by a speciation of the parasite produces a false cospeciation pattern [58,65,67]. If this process is common across the whole host-parasite association, a spurious cospeciation signal might be found when comparing topologies [39], and this may lead to an overestimation of cospeciation patterns by cophylogenetic methods [68]. To support temporal cospeciation, time must be taken into account, ideally from independent assessments of speciation time in hosts and their viruses over the time period. This is rarely possible because the inference for symbionts is generally made from host data [69]. Another approach is to rely on the estimation of molecular evolutionary rate in viruses to date speciation events [65]. Weaker, but nevertheless strong evidence, is provided the comparison of evolutionary divergence in cospeciating pairs. Such pairs ("copaths") are identified using methods such as Jane or TreeMap, because cospeciation events need to be inferred first. Copaths take into account the branch lengths connecting hosts and their cospeciating symbionts to these cospeciation events. If a correlation is found between copaths in hosts and corresponding viruses [70], i.e. via a significant linear regression when including all pairs, this supports cospeciation. In addition, showing that the intercept of this regression line is not different from 0 is again strong evidence for a cospeciation pattern. Whether or not such a pattern can be found depends on the cophylogenetic scenario considered (cospeciating pairs and corresponding copaths are different for each scenario), and as the number of scenarios is very high in the present host-virus system, it is not possible to study each of them to investigate if real cospeciation has taken place. The significant cospeciation signal observed here should then be considered with caution, and seems mainly due to the genus-level specificity. Nevertheless, the strength of the cophylogenetic signal observed in this analysis supports the hypothesis that virus evolution is in part driven by their hosts. If cospeciation is indeed happening within this host-virus association, it would imply that prasinoviruses evolve more slowly than their hosts, as [11] have shown, based on genomic data, that the evolutionary divergence between hosts is much higher than that between corresponding viruses. This host-virus system would then be a peculiar case among host-symbiont systems, where symbionts generally evolve faster than their hosts [9].

## Conclusion

The data and analyses provided in this paper support that prasinoviruses, while generally highly host specific, sometimes display a wide host range, with some strains able to infect hosts from different species. This can have important consequences when considering the role of viruses in microbial ecology.

A significant cospeciation signal between prasinoviruses and their hosts has been found in the cophylogenetic analyses performed in the present study, but their joint evolutionary history is complex, certainly involving host switches, duplication and losses, in addition to cospeciation events. Because it has been shown in a previous study that host genomes diverge more than corresponding viruses, additional data and analyses are needed to identify cospeciation events and to estimate the timing of these events, in order to be able to compare evolutionary rates in prasinoviruses and their hosts.

## Methods

### Hosts and viruses isolation

Hosts and viruses were isolated from environmental samples and kept in culture collections in Banyuls-sur-Mer and Roscoff. All hosts except *Ostreococcus lucimarinus* (CCE9901) and part of the viruses are referred to by their RCC (Roscoff Culture Collection) numbers (see Tables 2 and 3). Given the uncertainties concerning the species status of the genetic clades within *Micromonas, Ostreococcus* and *Bathycoccus*, we chose to use the currently accepted names *Micromonas pusilla* and *Bathycoccus prasinos* for all *Micromonas* and *Bathycoccus* strains respectively, and *Ostreococcus sp*., O. *lucimarinus, O. tauri* and *O. mediterraneus* for the strains belonging to the different *Ostreococcus* species. Prefixes Bp_, Mp_, and O_, Ot_ and Om_ were added to the RCC numbers to designate respectively *Bathycoccus prasinos*, *Micromonas pusilla*, and *Ostreococcus* sp.*, O. tauri* and *O. mediterraneus* strains (while O. *lucimarinus* is named Ol_CCE9901). Viruses from *Bathycoccus*, *Micromonas*, *Ostreococcus* are respectively named BatV or BpV, MicV or MpV, and OtV, OmV or OlV with numbers and letters corresponding to strains and the clade containing the host strain used for isolation. For example, MicAV31, refers to a *Micromonas* virus (strain 31) isolated from a clade A host.

Virus isolation and purification were obtained by a plating technique [7,13]. This method allowed us to visualize and pick off individual lysis plaques. Succinctly, seawater samples were filtered by gravity through membranes with a porosity of 3 μm then 0.45 μm. Filtrate were mixed with K-medium, growing host culture, a solution of hot agarose and poured in a Petri dish. Few days after plating, plaques appeared inside the agarose gel, they were picked off, mixed with 400 μl of a solution of MgSO_4_ (SM buffer; CSH Protocols; 2006; doi:10.1101/pdb.rec466) and conserved at 4°C. This technique ensures the presence of active viral particles in the isolate.

Isolation and growth of host strains was performed as in [18]. Briefly, seawater samples were mixed with Keller’s medium after filtration, and cultured for about 3 weeks. Cultures were then plated out to obtain individual clones on gel-solidified Keller’s medium or on L1 medium [71]. Colonies were then picked off for further growth after 3 weeks. Clonality was obtained on semi-solid agar plates: cells from the original strains were cultured in semi-solid agar K medium and individual colonies were picked off and transferred into new semi-solid agar medium. This process was repeated 2 or 3 times.

### Host specificity

We first assessed the pattern of host specificity, i.e. the host range of each viral strain investigated. Prasinoviruses infect hosts that can be cultured on plates [7], allowing host specificity to be tested experimentally [8]. We first tested the ability of each viral strain to grow on a plated clonal culture of each putative host strain, then kept for the subsequent analyses only host strains that supported growth of at least one viral strain. Plates of hosts were prepared (7 ml of K-medium, 8 ml of a 3.10^7^ cells/ml of a growing host culture and a 1.5% solution of agarose) and we added 2 μl of virus on the top of these plates. They were cultured (continuous light 100 μmol photon m^-2^ s^-1^, at 20 ± 1°C) inside a transparent plastic box to maintain humidity for 10 days. Plates that were not lysed 10 days after viral inoculation were considered not to be susceptible to infection by the virus.

Each test was performed in duplicate, to obtain a precise picture of the global pattern of host specificity in prasinoviruses. The specificity of 313 virus strains was tested on 26 host strains.

### Molecular data and phylogenetic reconstruction

Prasinoviruses are typically characterized by analyzing the sequence of a portion of the DNA polymerase gene (or dpo) [13,72]. This marker discriminates for viruses of all of the host genera investigated here, and was used to resolve their phylogenetic status. To amplify viral DNA polymerase fragments from lysis plaques we used a group of specific primers (AVS1-2-5) described previously [13,72]. Briefly, PCR reactions were set up as follows: 10 μl of virus lysis plaque liquid (with SM buffer) was added to a 90 μl reaction mixture which contained PCR assay buffer (Promega), 0.2 mM of each desoxyribonucleoside triphosphate, 1.5 mM MgCl_2_, 30 pmol of each primer and 0.5 U of Taq DNA polymerase (Promega). PCR bands were purified directly by using a nucleospin kit (Macheray-Nagel company) and DNA fragments were sequenced (Macrogen Inc., Korea or Genomic Core Facility (GENOMER) of the Station Biologique de Roscoff, France). To control for PCR or sequencing errors fragments were sequenced in reverse and forward directions and all nucleotide differences were checked visually.

Algal hosts were characterized via the sequencing of the full 18S rDNA (SSU) and Internal Transcribed Spacer 2 (ITS2). DNA was extracted by a modified cetyltrimethylammonium bromide (CTAB) protocol [73], and cells (200 ml of a dense culture) were harvested by centrifugation. The pellet was resuspended in 0.8 ml of CTAB buffer, incubated for 30 min at 60°C with 0.1 mg/ml proteinase K, and DNA was extracted by the addition of 0.8 mL of chloroform: isoamyl alcohol (24: 1). The sample was then gently agitated for 2 min, and the organic phase was removed after a 10 min centrifugation step at 4°C. The aqueous phase was recovered and incubated with 0.6 ml of isopropanol for 30 min at room temperature to precipitate the DNA. DNA was washed by the addition of 1 ml of EtOH 76%, dried, resuspended in sterile water and stored at - 20°C. Extracted DNA was used as a template to amplify the nuclear small subunit ribosomal and ITS2 genes. The eukaryotic primers Euk328f and Euk329r were used to amplify the 18S rDNA as described in [74] with the following conditions: an initial incubation step at 95°C for 5 min, followed by 34 cycles with a denaturing step at 95°C for 1 min, an annealing step at 62°C for 2 min and an extension step at 72°C for 3 min. These cycles were followed by a final extension step at 72°C for 7 min. The primers D1 (5′-GTA GGT GAA CCT GCG GAA GGA-3′), R1 (5′-CCTTGG TCC GTG TTT CTA GAC-3′), D2 (5′-ACC CGC CGA ATT TAA GCA TA-3′) and R2 5′-AGG GGA ATC CTT GTT AGT TTC-3′ were used to amplify the ITS1, 2 and 5.8S rDNA, with an initial incubation step at 94°C for 12 min, followed by 30 cycles with a denaturing step at 94°C for 1 min, an annealing step at 58°C for 2 min and an extension step at 72°C for 3 min. These cycles were followed by a final extension step at 72°C for 10 min. Polymerase chain reactions were carried out in an automated thermocycler (iCycler, Bio-Rad, Marne-la-Coquette, France). The PCR mixture (25 μl final volume) contained 2.5 μl of Mg Free Buffer 10X (1X final concentration, Promega, Madison, Wisconsin), 2.5 μl of MgCl_2_ solution (2.5 mM final concentration), 2 μl of deoxynucleoside triphosphate (dNTP, 400 μM final concentration each, Eurogentec), 0.5 μl of each primer (1 μM final contraction each), 0.125 μl of Taq Polymerase (5 units per μl, Promega, Madison, Wisconsin), sterile water and 1 μl of extracted DNA. PCR products were cloned using the TOPO TA cloning kit (Invitrogen, Carlsbad, CA, USA) following the protocol provided by the manufacturer. The 18S and ITS2 were concatenated (named here 18S + ITS) after their homogeneity was established using a partition homogeneity test [75].

We combined the results of the host specificity experimental assessment with molecular data to select a non-redundant virus dataset, and we kept the 51 virus strains differing in term of host specificity or dpo nucleotide sequence and the 22 host strains differing in their susceptibility to viruses or with differences in sequences. The full cross-infection dataset (313 virus strains on the 26 host strains) is available on request to the authors.

Sequences alignments were performed with MAFFT v5 [76,77], and ambiguously aligned regions were eliminated using GBlocks [78]. Phylogenetic reconstructions were based on DNA and amino acid (AA) sequences (for dpo), using Bayesian inference (BI) and maximum likelihood (ML). Evolutionary models were selected via Akaike Information Criterion using jModelTest v2 [79] for DNA sequences and ProtTest [80] for AA sequences. Bayesian analysis were carried out done with MrBayes 3.1.2 [81], with 4 chains of 10^6^ generations, trees sampled every 100 generations, and burnin value set to 20% of the sampled trees. In BI, coding DNA sequences (dpo) were considered with an evolutionary model designed for coding sequences taking the genetic code into account [82-84], and AA sequences were analyzed with a mixed model [81]. We checked that standard deviation of the split frequencies fell below 0.01 to ensure convergence in tree search. Maximum likelihood reconstructions were carried out using PhyML [85,86] and validated with 1000 bootstrap replicates.

No outgroups were used: trees were oriented using previous knowledge from [14] for hosts (where *Micromonas* is the basal lineage) and [11] for viruses (with BpV as the basal lineage).

### Cophylogeny

Several methods have been published to study cophylogenetic patterns between hosts and their symbionts [9,41], which can be classified into event-based methods and global fit methods [10]. Event-based methods aim at reconciling tree topologies of hosts and symbionts by adequately mixing generally four (sometimes more) kinds of coevolutionary events (cospeciation or codivergence, host-switch, duplication, sorting) and find the best reconstructions by minimizing its global cost (each event type is attributed a cost). A cophylogenetic scenario is produced, but the computational cost is very heavy (and the number of optimal scenarios can be very high), especially when exhaustive algorithms are used. The significance of the global cost is assessed against a random distribution of costs generated using random trees - if the observed optimal cost is significantly lower than optimal costs computed from randomly generated trees, then a global cospeciation signal is present. Global fit methods do not rely on events but assess the congruence between the two trees taking the pattern of host specificity into account encoded via a presence-absence matrix; again the observed level of congruence is tested against a random distribution. No scenario is produced but the computational burden is much lighter than for event-based methods, and a result can be obtained with any kind of associations, even with large trees and complex patterns of host specificity. We used an event-based method, Jane v4 [45] and a global fit method, ParaFit [43], implemented in CopyCat [87]. Jane was chosen (instead of the popular TreeMap [42], TreeMap 3 is currently being developed by Mike Charleston and is available at http://sites.google.com/site/cophylogeny, and was used here to draw the tanglegram on Figure 1) because it uses a heuristic algorithm that can be used even with complex host-symbiont systems such as this one. Jane considers a fifth type of coevolutionary event, "Failure to diverge", accounting for situation where, following a host speciation event, the symbiont remains on each new host species without speciating. Jane v4 can also handle polytomies (while TreeMap cannot). In Jane, polytomies are considered as soft polytomies, and the algorithm resolves polytomies in both trees in order to minimize the total cost of the reconstruction. The option "Prevent mid-polytomy" was selected to ensure the absence of duplication or host-switch involving the branch created to resolve the polytomy. In addition to assessing and testing the global congruence between trees, ParaFit can assess the contribution of each individual host-parasite association ("links") to this global congruence. This allows one to identify which host-parasite couples are the most structuring in the association. Jane was used with the following event-cost scheme (Cospeciation = 0, Duplication = 1, Host switch = 2, Sorting = 1, Failure to diverge = 1), a number of generations of 500 and a population size of 50. This cost scheme was used because we considered, as in several other studies, that cospeciation is the default situation, so its cost was set to 0 (e.g. [38,50,88]). Host-switching was considered as the least probable event and was assigned of cost of 2. For a good discussion on event costs, see [88]. Note that several cost schemes were assessed, and comparable results were obtained. Statistical tests for tree congruence in ParaFit and Jane were carried out with 999 permutations, and parasite trees instead of tip mappings were randomized in Jane.

### Availability of supporting data

GenBank accession numbers for the sequences used in this study are provided in Tables 2 and 3.

Sequences newly submitted for this study have GenBank accession numbers KF500985 to KF501037.

## Competing interests

The authors declare that they have no competing interests.

## Authors’ contributions

LB, CC, RE, EF and NS carried out the molecular biology and virology experiments. YD and LB performed the data analyses. YD, LB, NS and NG wrote the manuscript. All the authors read, edited and approved the final manuscript.
